# Network pharmacology combined with molecular docking and experimental validation to explore the potential mechanism of *Cinnamomi ramulus* against ankylosing spondylitis

**DOI:** 10.1080/07853890.2023.2287193

**Published:** 2023-11-29

**Authors:** Wendi Wei, Shaofeng Wu, Chenxing Zhou, Tianyou Chen, Jichong Zhu, Sitan Feng, Xinli Zhan, Chong Liu

**Affiliations:** The First Affiliated Hospital of Guangxi Medical University, Nanning, China

**Keywords:** *Cinnamomi ramulus*, ankylosing spondylitis, network pharmacology, immunohistochemistry, molecular docking

## Abstract

**Background:**

*Cinnamomi ramulus* (*C. ramulus*) is frequently employed in the treatment of ankylosing spondylitis (AS). However, the primary constituents, drug targets, and mechanisms of action remain unidentified.

**Methods:**

In this study, various public databases and online tools were employed to gather information on the compounds of *C. ramulus*, drug targets, and disease targets associated with ankylosing spondylitis. The intersection of drug targets and disease targets was then determined to identify the common targets, which were subsequently used to construct a protein-protein interaction (PPI) network using the STRING database. Network analysis and the analysis of hub genes and major compounds were conducted using Cytoscape software. Furthermore, the Metascape platform was utilized for Gene Ontology (GO) and Kyoto Encyclopedia of Genes and Genomes (KEGG) analyses. Molecular docking studies and immunohistochemical experiments were performed to validate the core targets.

**Results:**

The network analysis identified 2-Methoxycinnamaldehyde, cinnamaldehyde, and 2-Hydroxycinnamaldehyde as the major effective compounds present in *C. ramulus*. The PPI network analysis revealed PTGS2, MMP9, and TLR4 as the most highly correlated targets. GO and KEGG analyses indicated that *C. ramulus* exerts its therapeutic effects in ankylosing spondylitis through various biological processes, including the response to hormones and peptides, oxidative stress response, and inflammatory response. The main signaling pathways involved were IL-17, TNF, NF-kappa B, and Toll-like receptor pathways. Molecular docking analysis confirmed the strong affinity between the key compounds and the core targets. Additionally, immunohistochemical analysis demonstrated an up-regulation of PTGS2, MMP9, and TLR4 levels in ankylosing spondylitis.

**Conclusions:**

This study provides insights into the effective compounds, core targets, and potential mechanisms of action of *C. ramulus* in the treatment of ankylosing spondylitis. These findings establish a solid groundwork for future fundamental research in this field.

## 1. Introduction

Ankylosing spondylitis (AS) is a prevalent chronic inflammatory disorder characterized by joint inflammation and inflammation of ligament attachment points in the spine and pelvis, often resulting in chronic pain and mobility impairments [1]. Currently, AS remains incurable, and conservative therapy is the primary approach aimed at alleviating pain, improving joint function, reducing patient discomfort, and enhancing quality of life [2]. Western medicine treatments for AS involve the use of antirheumatic drugs, non-steroidal anti-inflammatory drugs (NSAIDs), glucocorticoids, and biological agents [3]. While these medications can mitigate pain and inflammation, they can also induce adverse drug reactions and side effects [4]. Therefore, there is an urgent need to identify safer and more effective therapeutic alternatives.

*Cinnamomi ramulus* (*C. ramulus,* Guizhi, GZ), a traditional Chinese medicine widely distributed in southern China, including Guangdong, Guangxi, and Yunnan [5], has been commonly used to treat AS in China [6]. It has demonstrated notable pain-relieving and joint function-improving effects in AS patients with minimal adverse reactions [7]. *In vivo* and *in vitro* pharmacological experiments have shown that *C. ramulus* possesses anti-inflammatory, antibacterial, antiviral, and antitumor properties, with its anti-inflammatory compounds offering new prospects for AS treatment [5]. However, few studies have explored the mechanisms through which *C. ramulus* exerts its therapeutic effects in AS, necessitating further investigation.

Network pharmacology, an interdisciplinary field merging network science, computer science, and bioinformatics, facilitates drug discovery and therapy by elucidating the interactions between drugs and disease-related genes, proteins, metabolites, and signaling pathways. Through network integration and analysis, it aids in the identification of novel drug targets and treatment strategies [8]. The present study focuses on investigating and analyzing the main compounds, core targets, and potential mechanisms underlying *C. ramulus* in the treatment of AS using network pharmacology. The findings are subsequently validated through molecular docking and immunohistochemical experiments, laying the theoretical foundation for future pharmacological research and clinical application.

## 2. Materials and methods

### 2.1. Active ingredient screening and target prediction for *C. ramulus*

*C. ramulus* was examined using the Traditional Chinese Medicine Systems Pharmacology Database and Analysis Platform (https://tcmsp-e.com/tcmsp.php) (accessed on 28 March 2023) [9]. The main active ingredients of the drug were selected based on criteria such as an ‘OB value ≥20%’, ‘DL value ≥0.1’, and adherence to Lipinski’s rule [10]. Targets associated with the effective compounds were obtained, and their protein names were converted to corresponding gene symbols using the UniProt database (https://www.uniprot.org/) (accessed on 28 March 2023). The effective compounds of *C. ramulus* were sourced from the Encyclopedia of Traditional Chinese Medicine (http://www.tcmip.cn/ETCM/index.php/Home/Index) (accessed on 28 March 2023) [11], with screening conditions based on the ‘Druglikeness Grading grade’ being categorized as ‘Moderate’ or ‘good’. The targets were collected and predicted using ETCM and SwissTargetPrediction (http://swisstargetprediction.ch/)(accessed on 28 March 2023) [12].

### 2.2. Prediction of disease targets associated with as

The disease targets associated with ankylosing spondylitis were gathered from various sources, including The Human Gene Database (https://www.genecards.org/), DisGeNET platform (https://www.disgenet.org/), Comparative Toxicogenomics Database (http://ctdbase.org/), and Gene Expression Omnibus(https://www.ncbi.nlm.nih.gov/) (GSE73754), using ‘Ankylosing Spondylitis’ as a keyword (all accessed on 18 March 2023). Subsequently, all the collected targets were merged, and duplicate entries were eliminated.

### 2.3. Screening of potential therapeutic targets

The drug targets of *C. ramulus* and the genes associated with AS were inputted into the online tool jvenn (https://jvenn.toulouse.inrae.fr/app/index.html) (accessed on 28 March 2023) [13] to identify the intersecting targets of *C. ramulus* and AS. Subsequently, the targets were further narrowed down to potential targets with therapeutic effects.

### 2.4. Establishment of PPI network maps of possible therapeutic targets

The possible targets were inputted into the STRING database (https://cn.string-db.org/) (accessed on 29 March 2023), with the species set as ‘Homo sapiens’ in the ‘organisms’ box. The initial protein-protein interaction (PPI) network was established using default parameters. TSV files were obtained from the STRING database and imported into Cytoscape 3.9.1 to visualize the PPI networks for the possible targets. The ClusterViz plug-in was utilized to further refine the selection of core genes. By applying the MCODE algorithm for analysis, the subnetwork with the highest score was identified, and all genes within that subnetwork were considered as the hub genes.

### 2.5. Establishment of effective compound-possible therapeutic targets network

The effective compounds and potential therapeutic targets were entered into Cytoscape 3.9.1 to create a network diagram illustrating the relationship between the effective compounds and the potential therapeutic targets. The network topology was analyzed, and the significance of the active ingredients was determined based on their degree values.

### 2.6. GO as well as KEGG analysis

The online gene annotation and analysis website Metascape (https://metascape.org/gp/index.html#/main/step1) (accessed on 2 April 2023) [14] was utilized to perform GO and KEGG analyses on the potential therapeutic targets. The identification of key GO terms and KEGG pathways was based on criteria such as ‘Min Overlap:3, p-Value Cutoff:0.01, Min Enrichment:1.5’. The enrichment results were then imported into a bioinformatics platform (https://www.bioinformatics.com.cn/) (accessed on 2 April 2023) [15] for further analysis and visualization of the data, and presented in the form of bubble maps.

### 2.7. Establishment of hub target-pathway network and herb-compound-hub target-pathway-disease network diagrams

The data pertaining to compounds, hub genes, and pathways were inputted into Cytoscape to create networks representing the connections between targets and pathways, as well as Chinese medicine compounds, targets, pathways, and diseases. These networks were then visualized for further analysis.

### 2.8. Molecular docking of key compounds to core targets

For The 3D structures of the core target proteins were obtained in PDB format from the Protein database RSCB Data Bank (https://www.rcsb.org/) (accessed on 1 May 2023) (PDB), while the 2D structures of the key compounds were obtained in SDF format from the PubChem chemical database (https://pubchem.ncbi.nlm.nih.gov/)(accessed on 3 April 2023). Ligand files and receptor files were prepared using SailVina (a script that interfaces with AutoDock Vina [16] and Open Babel [17] to perform batch) operations. Subsequently, molecular docking was conducted. The docking results were imported into OpenBabelGUI 3.1.1 and converted into pdb format files. PyMoL software was employed to visualize the docking results, and the visualized results were exported to pdb format and analyzed using the PLIP web tool (https://plip-tool.biotec.tu-dresden.de/plip-web/plip/index) [18] to identify receptor-ligand interactions.

### 2.9. Immunohistochemistry

In this study, a total of four patients with AS and kyphosis were selected from the First Affiliated Hospital of Guangxi Medical University as the experimental group. Additionally, four patients with lumbar fracture were included as the control group. Surgical resections of interspinous ligaments were collected from all participants. The surgical indication for AS patients was disabling kyphosis [19]. The control group consisted of patients presenting solely with lumbar fracture symptoms, without any systemic inflammatory or infectious conditions. Immunohistochemistry was conducted to compare the levels of core targets between the AS and control groups. Primary antibodies for MMP9 (Recombinant Anti-MMP9 antibody [RM1020] (ab283575)) and PTGS2 (Recombinant Anti-COX2/Cyclooxygenase 2 antibody [EPR12012] (ab179800)) were purchased from ABCAM (https://www.abcam.com/), and TLR4 (Anti-TLR4 Antibody BA1717) was obtained from BOSTER (https://www.boster.com.cn/). All the secondary antibodies (MaxVision TM HRP-Polymer anti-Mouse/Rabbit IHC Kit KIT-5020) were procured from MAXIM (http://maxim.com.cn/). The interspinous ligament samples underwent paraffin sectioning, deparaffinization, hydration, antigen retrieval, endogenous peroxidase inactivation, antibody incubation, DAB staining, sealing, and other laboratory procedures. A microscope was used for sample observation and image acquisition. The obtained immunohistochemical sections (24 in total) were analyzed for positivity using ImageJ software. The protein levels of the drug targets were compared between the AS and control groups using t-tests in IBM SPSS Statistics 27 software. Finally, statistical analysis of the results was performed using GraphPad Prism 9.5.

### 2.10. Statistical analysis

Data analysis was performed using ImageJ software and IBM SPSS Statistics 27 software. Inter-group differences were analyzed using the t-tests. A significance level of *p* < 0.05 was considered statistically significant.

## 3. Results

### 3.1. Compound and target collection and potential therapeutic target acquisition

**Figure 1.**
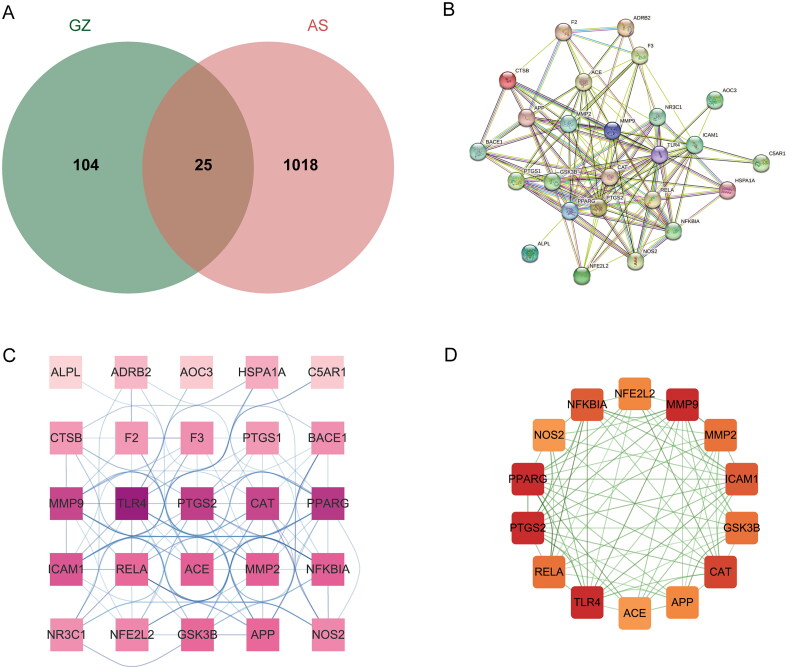
(A) The Venn diagram of overlapping targets of *C. ramulus* and as. Note: green areas represent drug targets, and red areas represent disease targets. (B, C) PPI network diagrams of the overlapping targets. (D) PPI network diagram of the hub targets.

A total of 26 drug compounds and 71 drug targets were obtained from the TCMSP database, while the ECTM database and SwissTargetPrediction yielded five drug compounds and 70 drug targets. After merging and removing duplicates, we obtained 31 compounds and 129 drug targets. Disease-associated targets were collected from the GeneCards database, DisGeNET database, CTD database, and GEO gene expression database (GSE73754). After integration and duplicate removal, a total of 1043 targets were obtained. By intersecting the *C. ramulus* drug targets with the AS disease-related targets, we identified 25 potential therapeutic genes. These genes were visualized using the online tool jvenn, resulting in a Venn diagram (Figure 1A). In the diagram, the green section represents the *C. ramulus* drug targets, the red section represents the AS disease-related targets, and the overlapping section in the middle represents the potential therapeutic targets.

### 3.2. PPI network was established for exploring hub targets

The 25 potential therapeutic targets were inputted into the String database to obtain PPI network data (Figure 1B). The PPI network data was then exported to Cytoscape software for constructing the PPI network (Figure 1C). The MCODE algorithm of the ClusterViz plug-in was employed for cluster analysis, resulting in the identification of a sub-network consisting of 14 hub targets with the highest score (Figure 1D). Based on the network topology parameters, the core targets with the highest degree were identified as PTGS2, MMP9, and TLR4.

### 3.3. Drug compound-potential therapeutic target network was constructed to explore the key compounds

**Figure 2.**
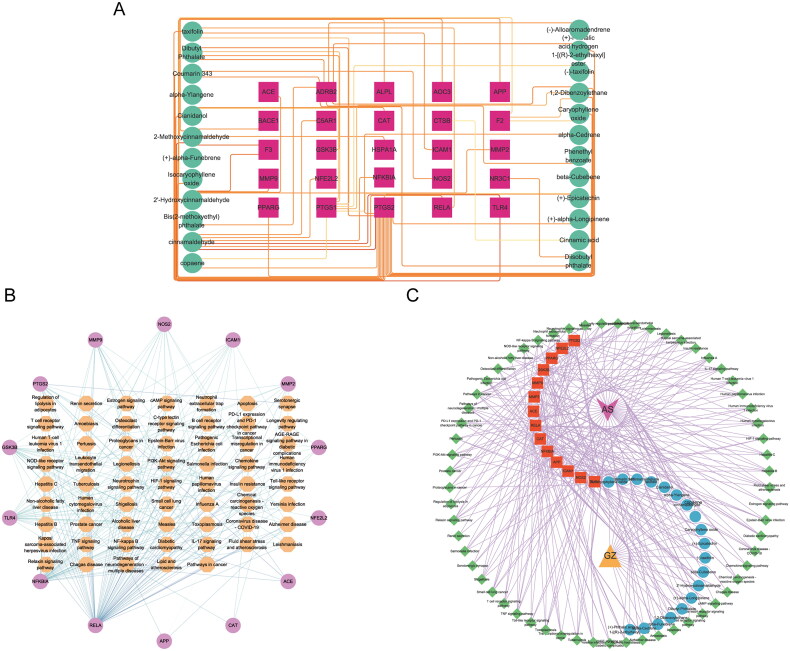
(A) Compound-overlapping target network diagram. Note: red squares represent overlapping targets, and green circles represent active compounds. (B) Pathway-hub target network diagram. Note: brown hexagons represent pathways, and purple circles represent hub targets. (C) Herb-compound-hub target-pathway-disease network diagram. Note: purple V-shape represents disease, yellow triangle represents herb, red squares represent hub targets, blue circles represent compounds, and green diamonds represent pathways.

A network was constructed to investigate the essential compounds of *C. ramulus* in treating AS by mapping 25 potential therapeutic targets and drug compounds using Cytoscape software. This network (Figure 2A), referred to as the ‘ Compound-overlapping target network diagram,’ provides insights into the mechanism of drug action and the interaction between drug compounds and biological targets. The drug compounds and targets were represented as nodes, and their interactions were depicted as edges. Network analysis was performed to identify key nodes based on their degree values. Among the top three compounds with the highest degree, 2-Methoxycinnamaldehyde, cinnamaldehyde, and 2 -Hydroxycinnamaldehyde were selected for further analysis (Table 1).

**Table 1.** Physiochemical properties, PubChem CID, 2D structure, and degree of core compounds.

Rank	Name	Molecular Formula	Molecular Weight	PubChem CID	Structure	Degree
1	2-Methoxycinnamaldehyde	C10H10O2	162.18 g/mol	641298	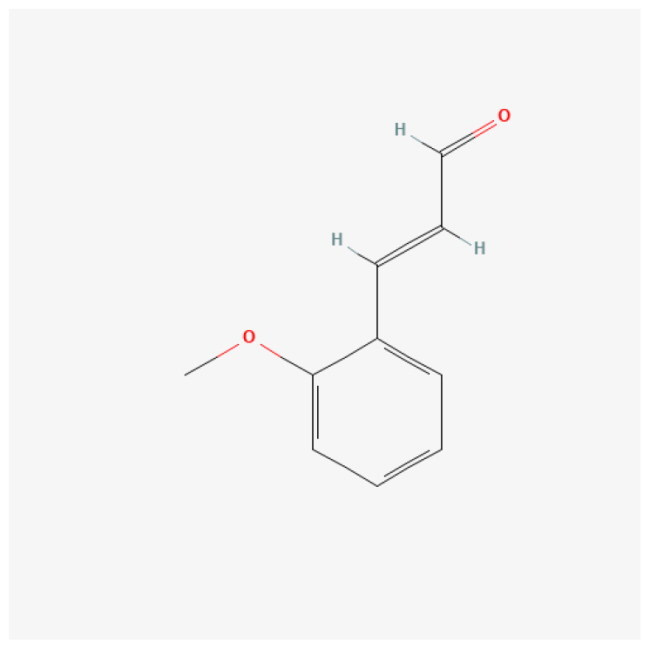	6
2	Cinnamaldehyde	C9H8O	132.16 g/mol	637511	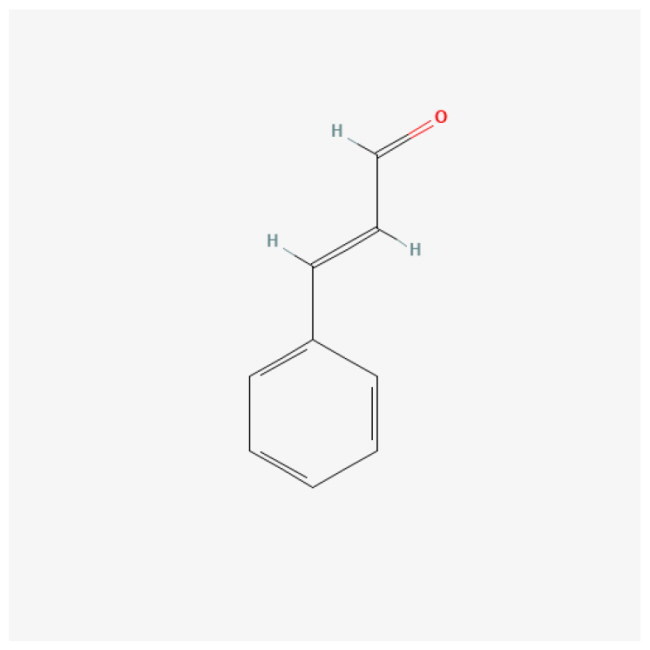	6
3	2-Hydroxycinnamaldehyde	C9H8O2	148.16 g/mol	5318169	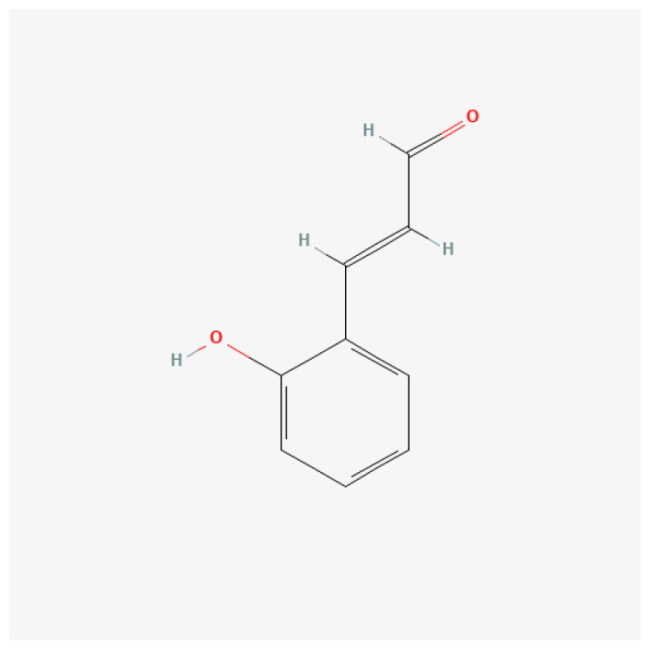	5

### 3.4. GO as well as KEGG pathway enrichment

**Figure 3.**
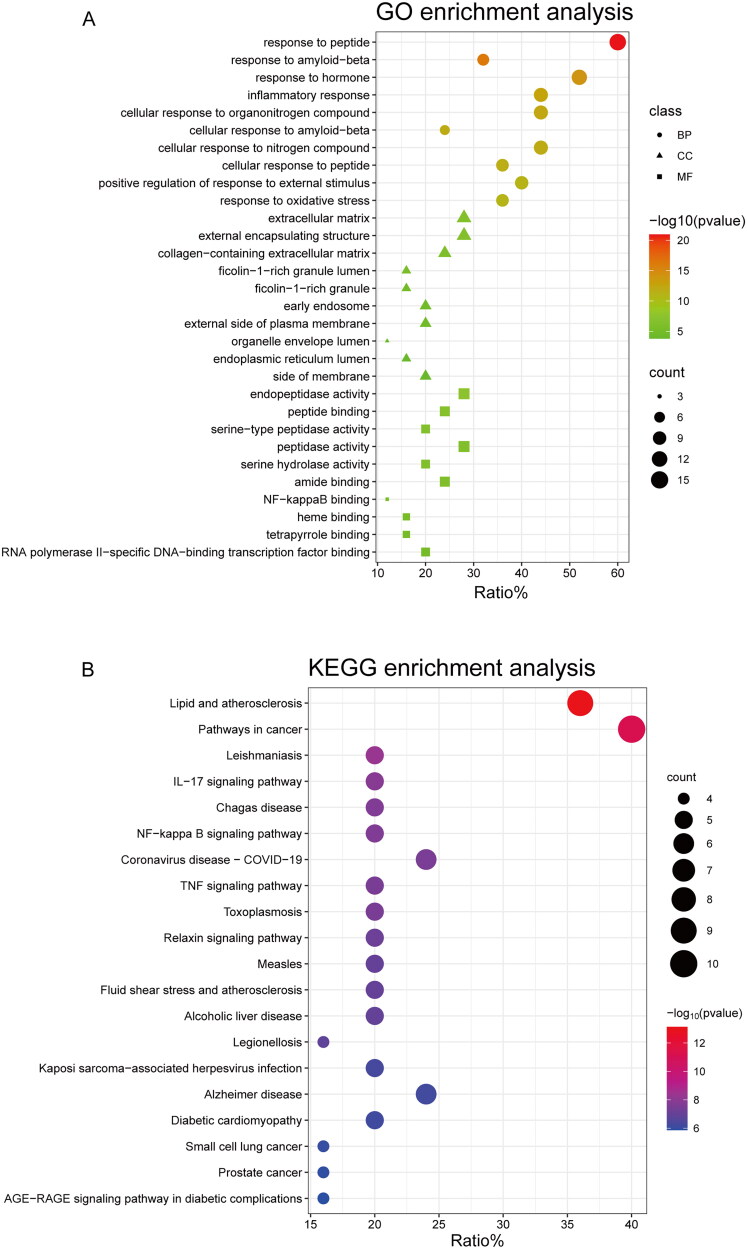
(A) Top 10 GO terms of overlapping genes. (B) Top 20 enriched KEGG pathways of overlapping genes.

In order to explore the functions associated with the 25 target genes shared by *C. ramulus* and AS, we conducted a systematic analysis to investigate the multiple mechanisms underlying the therapeutic effects of *C. ramulus* in AS treatment. GO enrichment analysis revealed 412 GO terms, including 360 biological process (BP), 22 cellular compound (CC), and 30 molecular function (MF) terms. The top 10 most significant BP, CC, and MF terms were selected as key results in GO annotation based on their p-value ranking. According to the BP analysis, these target genes are involved in various processes such as response to peptide, response to hormone, response to amyloid-beta, inflammatory response, cellular response to organonitrogen compound, cellular response to amyloid-beta, cellular response to nitrogen compound, cellular response to peptide, positive regulation of response to external stimulus, and response to oxidative stress. The CC analysis indicated that these target genes are primarily located in external encapsulating structures, extracellular matrix, collagen-containing extracellular matrix, ficolin-1-rich granules, ficolin-1-rich granule lumens, early endosomes, external sides of the plasma membrane, organelle envelope lumens, endoplasmic reticulum lumens, and sides of membranes. Based on the MF analysis, these target genes are mainly associated with functions such as endopeptidase activity peptide binding, peptidase activity and serine hydrolase activity, serine-type peptidase activity, amide binding, heme binding, NF-kappa B binding, tetrapyrrole binding, transcription factor binding, and RNA polymerase II-specific DNA-binding. These findings suggest that *C. ramulus* exerts its therapeutic effects primarily through the biological processes described above (Figure 3A). To identify potential pathways associated with *C. ramulus* in treating AS, KEGG enrichment analysis revealed 63 pathways related to AS. These pathways include IL-17, NF-kappa B, TNF, NOD-like receptor, Toll-like receptor, Chemokine, and PI3K-Akt pathways. The 20 most significant enrichment results were sorted based on their p-values in descending order. Based on these pathways, it is likely that the therapeutic effects of *C. ramulus* on AS are the result of the synergistic actions of multiple complex pathways (Figure 3B).

### 3.5. Exploration of major pathways responsible for the therapeutic effects of *C. ramulus* on as

The 14 hub targets were integrated with major *C. ramulus* compounds and AS-related pathways to generate network data. This data was then used to construct a ‘Pathway-hub target‘ network diagram in Cytoscape software (Figure 2B). The network diagram reveals a complex interplay between the hub targets and pathways. Through analysis of network parameters, it was observed that the TNF, IL-17, and NF-kappa B pathways, which are closely associated with inflammation, contain the highest number of core targets. These pathways are likely to be the most significant core pathways. The construction of the ‘Herb-compound-hub target-pathway-disease network diagram‘ network diagram allows for a comprehensive and systematic understanding of the intricate relationships and interactions between *C. ramulus* and AS (Figure 2C).

### 3.6. Molecular docking

**Figure 4.**
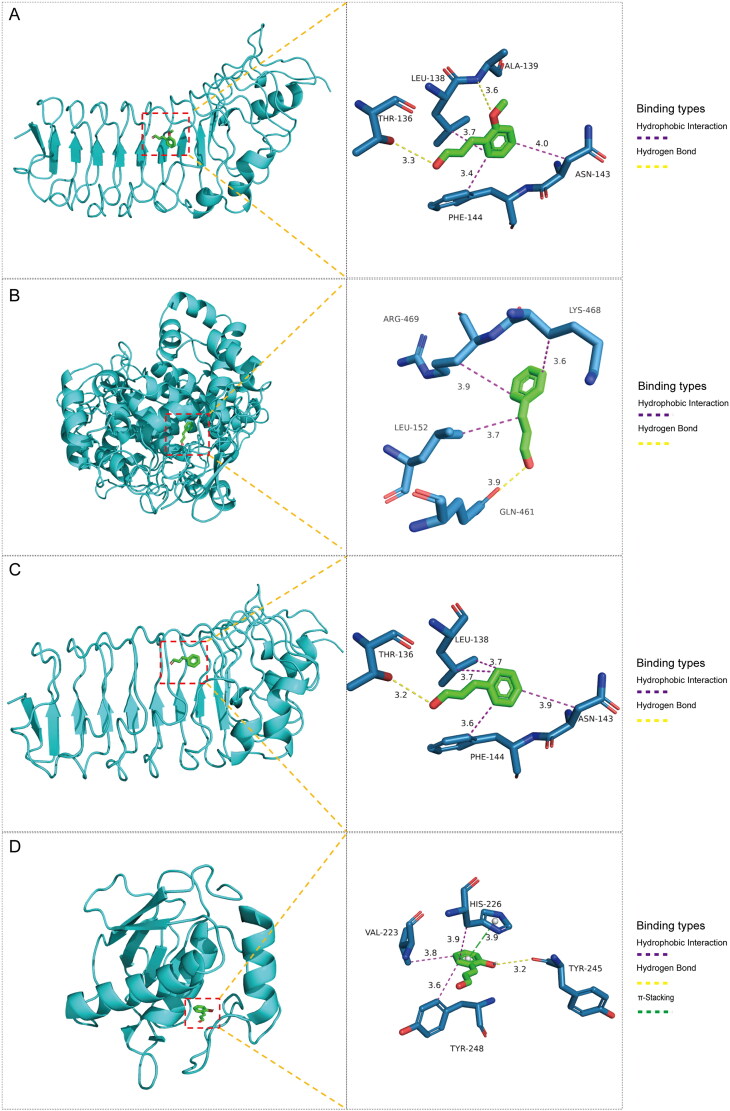
Molecular docking results of core compounds of *C. ramulus*: (A) 2-Methoxycinnamaldehyde - TLR4 (B) cinnamaldehyde - PTGS2 (C) cinnamaldehyde - TLR4 (D) 2-Hydroxycinnamaldehyde - MMP9.

To predict the binding of key drug compounds to the core targets, the three key compounds and three core targets with the highest degree values and network relationships were subjected to molecular docking. This approach calculated and simulated the interaction between these molecules to explore their binding patterns and capacities. The 3D structures of the three core genes: TLR4 (PDB: 3UL7), PTGS2 (PDB: 5F19), MMP9 (PDB: 6ESM) were obtained from the PDB database for the docking analysis. The best docking results for the three core genes and three bioactive compounds were visualized using 3D topology to demonstrate the compound-target binding model (Figure 4). Higher molecular docking scores indicate a stronger receptor-ligand binding ability while binding energy below −5 kcal/mol indicates favorable ligand-receptor binding. The ligands exhibited stable binding to their receptors, forming multiple binding modes at the active site, including hydrogen bonding, hydrophobic interaction, and π-stacking (Table 2). These findings highlight the interactions between the core targets and key compounds, which serve as the biological foundation for the multi-compound, multi-target action of *C. ramulus.*

**Table 2.** Binding energy of core compounds and core targets.

Ligands	Receptors	PDB ID	Binding types	Binding energy (kcal/mol)
2-Hydroxycinnamal­-dehyde	MMP9	6ESM	Hydrophobic Interactions, Hydrogen Bonds, π-Stacking	–7.4
Cinnamaldehyde	PTGS2	5F19	Hydrophobic Interactions, Hydrogen Bonds	–5.6
Cinnamaldehyde	TLR4	3UL7	Hydrophobic Interactions, Hydrogen Bonds	–6.6
2-Methoxycinnamal­-dehyde	TLR4	3UL7	Hydrophobic Interactions, Hydrogen Bonds	–7.5

### 3.7. The expression of core targets was verified by immunohistochemistry

**Figure 5.**
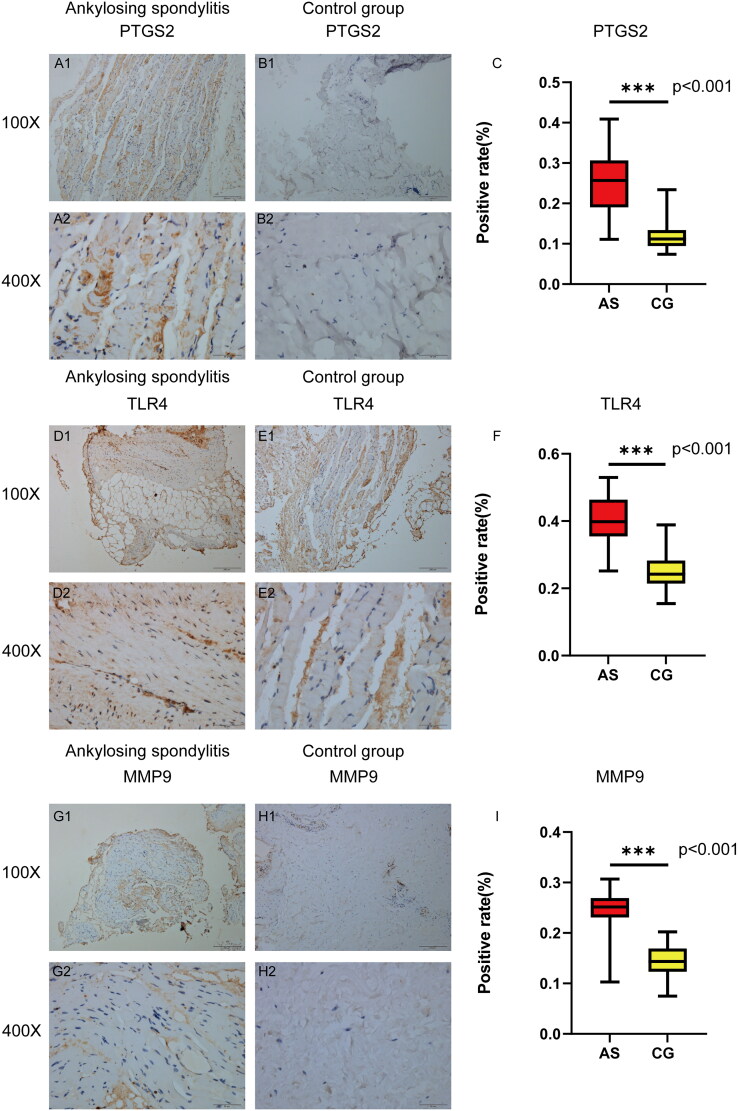
Immunohistochemical staining analysis. (A, B, D, E, G, H) show the specific expression of PTGS2, TLR4, MMP9 in the as group and the control group. (C, F, I) show the statistical analysis results of the positivity rate between the as group and the control group.

Immunohistochemical staining was conducted on tissue samples obtained from four AS patients and four control patients to examine the levels of PTGS2, MMP9, and TLR4. It is evident that the levels of PTGS2, MMP9, and TLR4 in the AS group were significantly higher compared to the control group (Figure 5). The IHC images were analyzed using ImageJ software, and the expression data of the target proteins were imported into SPSS 27.0 for statistical analysis. The independent sample t-test was used to assess inter-group differences. The results revealed a significant increase in the positive rates of PTGS2, MMP9, and TLR4 in the AS group compared to the control group (*p* < 0.05). This finding confirms the differential expression of PTGS2, MMP9, and TLR4 between the AS and control groups, thereby validating our hypothesized core targets.

## 4. Discussion

Through the analysis of the PPI network, we identified PTGS2, MMP9, and TLR4 as the three most crucial targets. PTGS2 encoding the COX-2 enzyme, plays a critical role in the inflammatory response as a prostaglandin synthetase [20]. The expression of PTGS2 significantly increases in ankylosing spondylitis cases [21]. PGE2 acting through the prostaglandin E (EP) receptor, modulates T cell activation, differentiation, maturation, secretion of factors (such as IL-17, TNF-α), and participates in inflammation [22]. Selective COX-2 inhibitors have been clinically used to alleviate pain and disease activity in AS [23]. MMP9 encoded by the matrix metalloproteinase 9 gene, can degrade certain proteins in the extracellular matrix and promote the infiltration of inflammatory cells [24]. Previous studies have demonstrated an elevation in plasma MMP9 expression among AS patients, which is associated with disease duration and the severity of the inflammatory response [25]. In animal experiments, inhibition of MMP-9 has been shown to reduce the inflammatory response in AS mouse models [26]. TLR4, a receptor protein involved in regulating immune and inflammatory responses, is also implicated in AS-related pathological osteogenesis [27]. Intestinal bacteria-derived lipopolysaccharide (LPS) has been found to activate monocytes and macrophages through TLR4, enhancing the HLA B27 fold protein response and inducing the release of inflammatory cytokines [28]. LPS combined with TLR4 promotes the growth and osteogenic differentiation of adipose tissue-derived mesenchymal stem cells (adMSC) by activating the NF-kB signaling pathway. Conversely, inhibiting TLR4 competitively suppresses LPS-mediated adMSC proliferation and osteogenic differentiation [29]. In summary, PTGS2, MMP9, and TLR4 levels are closely associated with AS pathology.

In the Compound-overlapping target network, we identified cinnamaldehyde, 2-Methoxycinnamaldehyde, and 2-Hydroxycinnamaldehyde as the most significant active ingredients. Cinnamaldehyde exhibits anti-inflammatory activity and is a characteristic *C. ramulus* compound [5]. In cellular experiments, cinnamaldehyde has been shown to downregulate LPS-mediated TNF-α, IL-1β, and IL-6 levels in human synoviocytes, inhibit the TLR4 signaling pathway, and significantly reduce synoviocyte inflammation [30]. Additionally, cinnamaldehyde reduces mesenteric I/R injury in rats by downregulating cox-2 levels and inhibiting the NF-kB pathway [31]. As an anti-inflammatory agent, 2-Methoxycinnamaldehyde can mitigate liver injury caused by ischemia-reperfusion injury in rats by decreasing TLR4 expression, inhibiting NF-kB activation, and reducing the release of inflammatory mediators such as TNF-α and IL-1β [32]. Moreover, 2-Hydroxycinnamaldehyde efficiently suppresses TNF-α-induced MMP9 expression in human aortic smooth muscle cells [33]. These key compounds are closely linked to the core targets. Through the network relationships, it can be inferred that *C. ramulus* may synergistically exhibit anti-inflammatory and immunomodulatory effects by acting on multiple targets through its various compounds.

GO annotation analysis revealed the involvement of various biological processes, including the response to hormones/peptides, inflammatory response, and oxidative stress response. Previous studies have suggested a negative correlation between 1, 25-dihydroxyvitamin D3 and AS, indicating a potential protective effect on AS [34]. Furthermore, specific peptides such as FGA-peptide (sequences: DSGEGDFLAEGGGVRGPR), TUBB-peptide (sequences: ISEQFTAMFR), and C4A-peptide (sequences: NGFKSHAL) have been found to significantly enhance fibroblast growth in AS patients [35]. Inflammatory response is a key feature of ankylosing spondylitis, as chronic inflammation plays a crucial role in disease progression and aggravation. Prolonged oxidative stress can lead to cellular damage and exacerbate inflammation [36]. NF-kB activation and overexpression are closely associated with inflammatory response and tissue damage in ankylosing spondylitis [37]. These responses have significant implications for the onset and progression of the disease.

**Figure 6.**
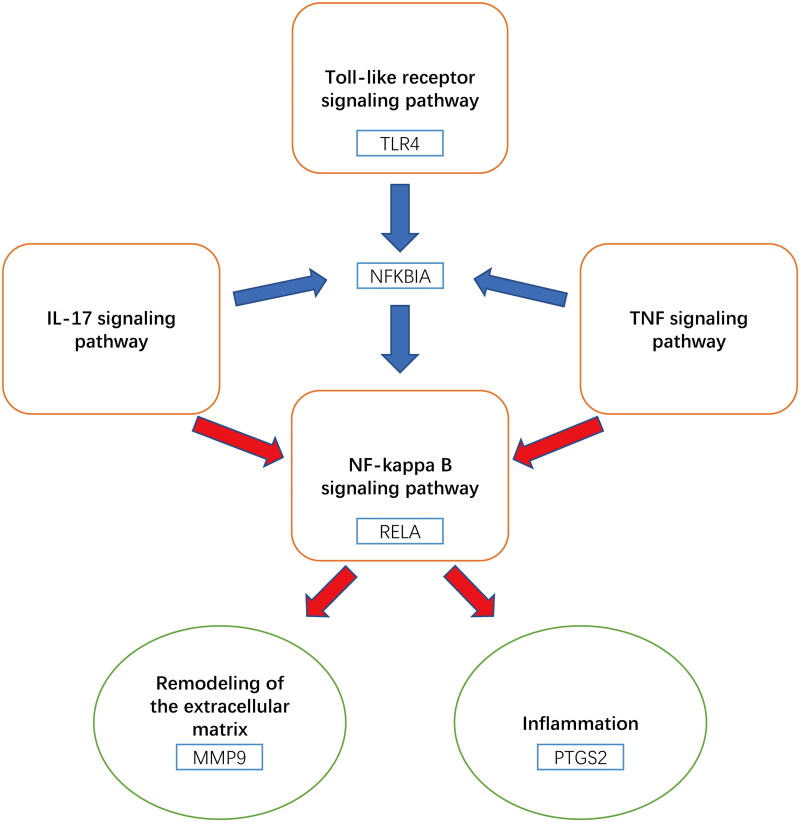
Core targets and potential mechanism of key compounds in *C. ramulus* against as.

KEGG analysis identified multiple pathways closely related to AS pathology. Network analysis highlighted IL-17, TNF, NF-kappa B, and Toll-like receptor pathways as the main pathways involved (Figure 6). In AS cases, there is an increase in the number and activity increase of Th17 cells, leading to overexpression of IL-17 [38]. Injection of IL-17 in a mouse model of arthritis resulted in increased levels of COX-2 and MMP-9 in synoviocytes [39]. Inhibiting the IL-17 signaling pathway has emerged as a novel therapeutic approach for AS, with agents targeting this pathway effectively reducing the inflammatory response and alleviating symptoms in AS patients [40]. Activation of the NF-KB pathway significantly increased in AS patients, and the expression of TLR4 gene, associated with AS, is also elevated [41]. In a rat model of ischemia-reperfusion, cinnamaldehyde-mediated inhibition of the NF-kB signaling pathway downregulates the expression of COX-2 and other inflammatory factors, thereby reducing cell apoptosis and inflammatory response [31]. The binding of TNF to TNFR1 leads to local inflammation, which is further amplified by mitogen-activated kinase (MAPK) and NF-kB activation, resulting in damage to bone, intervertebral discs, and cartilage in AS [42]. TNF promotes osteogenic differentiation by inducing the expression of bone morphogenetic proteins (BMPs), Osterix, RunX2, and alkaline phosphatase (ALP) through the activation of the NF-kB pathway [43]. Drugs targeting TNF-α have been widely used in the treatment of AS with positive outcomes [44]. Some experiments have demonstrated elevated levels of TLR4 and TLR5 in peripheral blood mononuclear cells of AS patients, while TLR3 levels were decreased. Treatment with TNF-α inhibitors, led to an increase in TLR3 levels, inhibition of NF-kB activation, and a decrease in TLR4 and TLR5 levels [45]. Inhibition of the Toll-like receptor pathway, specifically TLR4, has shown promising results in reducing disease activity in AS by suppressing downstream NF-kB activation [41].

The molecular docking analysis revealed that the active compounds of *C. ramulus* exhibited a strong binding affinity to the core targets, indicating a high likelihood of these compounds interacting with the core targets. Immunohistochemistry results further supported this finding by demonstrating differential expression of the predicted core targets, namely PTGS2, MMP9, and TLR4, in AS patients. The expression levels of PTGS2, MMP9, and TLR4 were upregulated, which is consistent with previous reports. These findings not only validate the reliability of the network analysis but also confirm the accuracy of the drug target prediction.

However, this study has certain limitations. The results obtained from network pharmacology, molecular docking, and immunohistochemistry are preliminary. Therefore, it is necessary to conduct pharmacological and molecular biology experiments to further validate the research findings.

## 5. Conclusion

Based on network pharmacology and immunohistochemistry experiments, this study provides initial insights into the potential mechanism of action of *C. ramulus* in treating AS. The key compounds identified, namely cinnamaldehyde, 2-Methoxycinnamaldehyde, and 2-Hydroxycinnamaldehyde, may play a crucial role in the therapeutic effects of *C. ramulus*. The core targets identified, PTGS2, MMP9, and TLR4, may be involved in regulating important pathways such as IL-17, TNF, NF-kB, and Toll-like receptor pathways. The validity of these findings was confirmed through molecular docking and immunohistochemistry analyses. Overall, *C. ramulus* may exert its therapeutic effects by modulating various inflammatory factors, signaling pathways, and biological processes, leading to the inhibition of inflammatory response and regulation of immunity. These findings contribute to a better understanding of the underlying mechanisms of *C. ramulus* in treating ankylosing spondylitis and provide a foundation for developing new therapeutic strategies.

## Supplementary Material

Supplemental Material

## Data Availability

All data collected in the present work are obtained from public databases, and part of the data are presented in Supplementary material.
