# Supplementary material for: Network pharmacology combined with molecular docking and experimental validation to explore the potential mechanism of Cinnamomi ramulus against ankylosing spondylitis
Source: Ann Med. 2023 Nov 29;55(2):2287193. doi: 10.1080/07853890.2023.2287193 (PMC10836281; doi:10.1080/07853890.2023.2287193)
Supplement: Supplemental Material [file IANN_A_2287193_SM9962.docx]

**Table S1 Network parameters for core targets**

| name | Closeness  Centrality | Clustering  Coefficient | Degree | Neighborhood  Connectivity | Radiality | Stress | Topological  Coefficient |
| --- | --- | --- | --- | --- | --- | --- | --- |
| PTGS2 | 1 | 0.794871795 | 26 | 10.53846154 | 1 | 32 | 0.810650888 |
| MMP9 | 1 | 0.794871795 | 26 | 10.53846154 | 1 | 32 | 0.810650888 |
| TLR4 | 1 | 0.794871795 | 26 | 10.53846154 | 1 | 32 | 0.810650888 |
| PPARG | 0.928571429 | 0.803030303 | 24 | 10.66666667 | 0.994083 | 26 | 0.820512821 |
| CAT | 0.928571429 | 0.803030303 | 24 | 10.66666667 | 0.994083 | 26 | 0.820512821 |
| ICAM1 | 0.866666667 | 0.836363636 | 22 | 10.90909091 | 0.988166 | 18 | 0.839160839 |
| NFKBIA | 0.866666667 | 0.872727273 | 22 | 11.09090909 | 0.988166 | 14 | 0.853146853 |
| MMP2 | 0.8125 | 0.888888889 | 20 | 11.3 | 0.982249 | 10 | 0.869230769 |
| GSK3B | 0.8125 | 0.888888889 | 20 | 11.3 | 0.982249 | 10 | 0.869230769 |
| RELA | 0.8125 | 0.844444444 | 20 | 11 | 0.982249 | 14 | 0.846153846 |
| APP | 0.764705882 | 0.888888889 | 18 | 11.33333333 | 0.976331 | 8 | 0.871794872 |
| NFE2L2 | 0.764705882 | 0.944444444 | 18 | 11.77777778 | 0.976331 | 4 | 0.905982906 |
| ACE | 0.722222222 | 0.964285714 | 16 | 11.75 | 0.970414 | 2 | 0.903846154 |
| NOS2 | 0.722222222 | 0.964285714 | 16 | 12 | 0.970414 | 2 | 0.923076923 |

**Table S2 Network parameters for core components**

| name | Betweenness  Centrality | Closeness  Centrality | Degree | Neighborhood  Connectivity | Radiality | Stress | Topological  Coefficient |
| --- | --- | --- | --- | --- | --- | --- | --- |
| 2-Methoxycinnamaldehyde | 0.376812 | 0.267442 | 6 | 1.333333 | 0.838875 | 1360 | 0.166666667 |
| cinnamaldehyde | 0.497902 | 0.370968 | 6 | 4 | 0.900256 | 1972 | 0.176470588 |
| 2'-Hydroxycinnamaldehyde | 0.168116 | 0.188525 | 5 | 1.2 | 0.746803 | 580 | 0.2 |
| 1,2-Dibenzoylethane | 0.122305 | 0.333333 | 4 | 7.2 | 0.882353 | 834 | 0.31 |
| taxifolin | 0.064842 | 0.306667 | 4 | 6.75 | 0.867008 | 432 | 0.359375 |
| Dibutyl Phthalate | 0.099492 | 0.328571 | 4 | 8.25 | 0.879795 | 694 | 0.3625 |
| Cianidanol | 0.046802 | 0.302632 | 3 | 8.333333 | 0.86445 | 232 | 0.458333333 |
| Coumarin 343 | 0.085604 | 0.323944 | 3 | 8.666667 | 0.877238 | 520 | 0.383333333 |
| (+)-Phthalic acid hydrogen 1-[(R)-2-ethylhexyl] ester | 0.042126 | 0.319444 | 2 | 12.5 | 0.87468 | 396 | 0.575 |
| Caryophyllene oxide | 0.010163 | 0.298701 | 2 | 10 | 0.861893 | 72 | 0.5625 |
| Diisobutyl phthalate | 0.043478 | 0.223301 | 2 | 4.5 | 0.795396 | 328 | 0.5 |
| (+)-Epicatechin | 0.003324 | 0.298701 | 2 | 12 | 0.861893 | 54 | 0.6875 |
| Isocaryophyllene oxide | 0.010163 | 0.298701 | 2 | 10 | 0.861893 | 72 | 0.5625 |
| (1R,2R,6R,7S,8S)-1,3-dimethyl-8-propan-2-yltricyclo[4.4.0.02,7]dec-3-ene | 0.003324 | 0.298701 | 2 | 12 | 0.861893 | 54 | 0.6875 |
| (-)-taxifolin | 0.003324 | 0.298701 | 2 | 12 | 0.861893 | 54 | 0.6875 |
| Cinnamic acid | 0 | 1 | 1 | 1 | 1 | 0 | 0 |
| (+)-alpha-Funebrene | 0 | 0.294872 | 1 | 17 | 0.859335 | 0 | 0 |
| alpha-Ylangene | 0 | 0.294872 | 1 | 17 | 0.859335 | 0 | 0 |
| (-)-Alloaromadendrene | 0 | 0.221154 | 1 | 8 | 0.792839 | 0 | 0 |
| beta-Cubebene | 0 | 0.294872 | 1 | 17 | 0.859335 | 0 | 0 |
| (+)-alpha-Longipinene | 0 | 0.294872 | 1 | 17 | 0.859335 | 0 | 0 |
| alpha-Cedrene | 0 | 0.294872 | 1 | 17 | 0.859335 | 0 | 0 |
| Phenethyl benzoate | 0 | 0.221154 | 1 | 8 | 0.792839 | 0 | 0 |
| Bis(2-methoxyethyl) phthalate | 0 | 0.221154 | 1 | 8 | 0.792839 | 0 | 0 |
